# Efficacy of Adjuvant Tamsulosin for Improving the Stone-Free Rate after Extracorporeal Shock Wave Lithotripsy in Renal Stones: A Randomized Controlled Trial

**DOI:** 10.1155/2022/3757588

**Published:** 2022-01-31

**Authors:** Rafael Edgardo Maldonado-Valadez, Angel David Valdez-Vargas, José Antonio Alvarez, Edel Rafael Rodea-Montero

**Affiliations:** ^1^Department of Urology, Hospital Regional de Alta Especialidad Del Bajío, Leon, Mexico; ^2^Universidad de Guanajuato, Leon, Mexico; ^3^Department of Research, Hospital Regional de Alta Especialidad Del Bajío, Leon, Mexico

## Abstract

**Introduction:**

Extracorporeal shock wave lithotripsy (ESWL) is an effective treatment for urolithiasis. Tamsulosin is capable of causing dilation and facilitating the migration of stones. The aim of this study is to evaluate the efficacy of adjuvant treatment with tamsulosin for improving the stone-free rate after a single session of ESWL in the treatment of kidney stones.

**Methods:**

This is a randomized, nonplacebo-controlled study with a sample of 60 adults with a single radiopaque kidney stone of 5–20 mm in diameter. After the ESWL session, the patients were divided into two groups. The control group received standard treatment for analgesia consisting of oral diclofenac (75 mg/12 h) as needed. The tamsulosin group received standard treatment for analgesia plus oral tamsulosin (0.4 mg/day) for eight weeks. In both groups, stone-free status was determined using a CT scan eight weeks after ESWL. The protocol of this study was registered with ClinicalTrials.gov, identifier: NCT04819828.

**Results:**

Only 57 patients completed the study (28 tamsulosin and 29 control). Overall, the average stone diameter was 11.42 ± 4.52 mm. The stone-free rate was 50.88% (29 of 57) overall, 53.57% (15 of 28) for the tamsulosin group, and 48.27% (14 of 29) for the control group (*p* = 0.680). The estimated relative risk in favor of the tamsulosin group to achieve a stone-free status was 1.11 (95% CI 0.67–1.9). The estimated number needed to treat to achieve a single patient with renal stone-free status after eight weeks of ESWL adjuvant treatment with tamsulosin was 19.

**Conclusion:**

Our findings suggest that tamsulosin as adjuvant treatment after a single ESWL session is well tolerated and safe, but it does not increase the stone-free rate in patients with a single radiopaque renal stone of 5–20 mm in diameter. Our results may support the use of tamsulosin with ESWL in the case of patients with a single radiopaque renal stone of 11–20 mm in diameter based on an apparent higher stone-free rate and a low rate of complications.

## 1. Introduction

Urolithiasis is a common health problem worldwide [1], affecting ∼10% of the population at some stage in their lives [2]. It affects approximately 5% of women and 12% of men in the United States, and it has been suggested that the incidence is increasing [3]. Because of its efficacy and low morbidity, extracorporeal shock wave lithotripsy (ESWL) is an effective treatment for kidney stones smaller than 20 mm in diameter [4]. The objective of this therapy is to achieve adequate fragmentation of the calculus that allows spontaneous expulsion of the fragments, and finally a stone-free state, which is not always possible [5].

The presence of adrenergic receptors in the ureter has suggested the involvement of the sympathetic nervous system in its peristaltic activity [6, 7]. It has also been shown that alpha 1 adrenergic antagonist medications such as tamsulosin are capable of inhibiting basal tone and ureteral peristalsis, causing dilation and facilitating the migration of stones [8]. Some authors have reported the efficacy of this type of medication for spontaneous calculus expulsion [9, 10], but there is no conclusive evidence of the adjuvant effectiveness of tamsulosin after ESWL for stone clearance and even less among a Mexican population.

The aim of this study was to evaluate the efficacy of adjuvant treatment with tamsulosin for improving the stone-free rate after a single session of ESWL in the treatment of radiopaque kidney stones.

## 2. Materials and Methods

### 2.1. Patients

All of the participants were Mexican-Hispanic and were recruited prospectively between January 2010 and June 2016 from consecutive patients attending the Urology Department at the tertiary care Mexican High-Specialty Regional Bajio Hospital (Hospital Regional de Alta Especialidad del Bajío, HRAEB), located in León City in Guanajuato State (Mexico). A total of 362 patients (men and women ≥18 years old) with a single radiopaque kidney stone of 5–20 mm in diameter and visible on a computed tomography (CT) scan of the abdomen were recruited during the screening phase. Of these, 293 were excluded based on the study criteria and nine refused to participate. The remaining 60 patients were enrolled and randomly divided into two groups: 30 were assigned to the control group and 30 to the tamsulosin group.

The exclusion criteria were lower calyx stones; a history of spontaneous stone passage; a previously failed ESWL; treatment with alpha adrenergic antagonists, calcium channel inhibitors, or steroids; severe obesity (BMI ≥ 40); pregnancy; serum creatinine ≥2 mg/dl; renal artery aneurysm and/or abdominal aorta aneurysm; the presence of a ureteral stent; anatomical abnormalities or previous surgery on the upper urinary tract; bone deformities; the presence of a urinary tract infection; coagulation disorders; or poorly controlled hypertension.

## 3. Methods

The protocol of this study was registered with ClinicalTrials.gov, identifier: NCT04819828. All methods were performed in accordance with the relevant guidelines and regulations.

We conducted a randomized, nonplacebo-controlled study with a sample of 60 adults (men and women ≥18 years old). Data regarding age, serum creatinine, cell blood count, coagulation tests, and urine culture were obtained from the clinical records of the patients, and the result of a pregnancy test was included in the case of women. Before treatment with ESWL, the diagnostics of a single radiopaque kidney stone of 5–20 mm in diameter and the characteristics of the renal calculus, such as its laterality (left/right), location (renal pelvis, upper calix, or middle calix), and diameter (mm), were determined using abdominal radiography evaluating the kidneys, ureter, and bladder (KUB) accompanied by CT of the abdomen.

The ESWL session was carried out on an outpatient basis with the Siemens Lithostar Modularis lithotripter (Siemens, Erlangen, Germany) using electromagnetic energy and monitored by fluoroscopy. All patients were placed under general anesthesia based on propofol (1 mg/kg of weight), midazolam (50 *µ*g/kg of weight), and fentanyl (1 *µ*g/kg of weight). The session was finished upon reaching a maximum of 4,000 shocks, or if complete, fragmentation of the kidney stone was observed.

At the end of the ESWL session, the patients were divided into two groups (the control group and the tamsulosin group) using a table of random numbers. After discharge, all patients were instructed to drink a minimum of 2 L of water daily. The control group received standard treatment for analgesia consisting of oral diclofenac (75 mg/12 h) as needed. The tamsulosin group received standard treatment for analgesia plus oral tamsulosin (0.4 mg/day) for eight weeks.

Patients attended follow-up visits every two weeks during the first month of treatment and a final visit at the end of the second month. During each visit, vital signs were taken, a physical examination was conducted, and possible adverse effects were monitored; additionally, a plain X-ray of the KUB was taken at two and four weeks to evaluate possible complications associated with residual fragments [11–13], as well as an abdominal CT scan eight weeks after ESWL to determine stone-free status.

In both groups, the efficacy of ESWL therapy and treatment (stone-free status) was determined based on the absence of residual stones (≥5 mm in diameter), the presence of asymptomatic nonsignificant residual stone fragments (≤4 mm in diameter), and the absence of additional procedures to resolve an event of acute symptomatic urinary obstruction, as recommended by the European Association of Urology Working Group Panel in the Guidelines on Urolithiasis 2010 [14]. The results were determined by a urologist who did not know which treatment group the patients had been assigned to.

### 3.1. Sample Size

In this study, sample size calculations were performed based on the literature-reported incidence of a stone-free rate after ESWL of approximately 60% (0.60) and an expected difference from adjuvant tamsulosin treatment of approximately 30% (0.90) [11, 15] with an alpha level of 0.05 and a beta level of 0.20 (80% potency) with a unilateral hypothesis. A total of 25 patients per group were required, but due to probable losses during follow-up, 30 patients per group were included.

### 3.2. Statistical Analysis

Data were analyzed using R statistical software [16]. Descriptive statistics were determined for the patients' clinical characteristics, grouped by the treatment assigned (control group and tamsulosin group), and compared using the Mann–Whitney *U* test or the chi-square test depending on the variable type. The strength of the association between ESWL treatment with adjuvant tamsulosin and the stone-free rate was evaluated by calculating the relative risk and the number needed to treat. In all cases, alpha = 0.05 was considered significant.

## 4. Results

**Figure 1:**
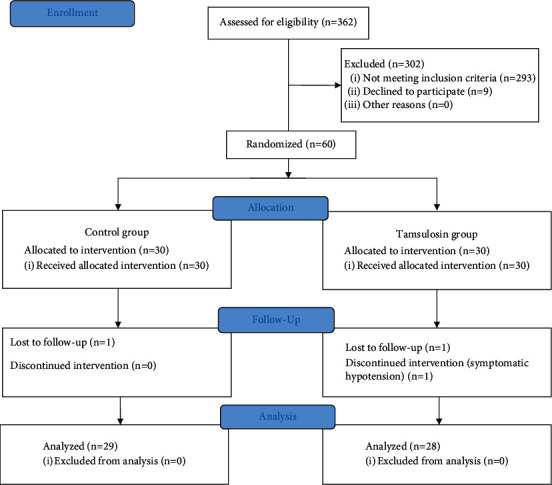
CONSORT (Consolidated Standards of Reporting Clinical Trials) flow diagram of patient progress during the phases of the randomized trial.

A total of 60 adult patients (men and women 20–76 years old) with a single radiopaque kidney stone between 5 and 20 mm in diameter were enrolled and randomly divided into two groups: 30 were assigned to the control group and 30 to the tamsulosin group. During the follow-up period, treatment was suspended for one patient who presented with adverse effects characterized by symptomatic hypotension in the tamsulosin group (1 of 30; 3.33%). In addition, one patient in the tamsulosin group (1 of 30; 3.33%) and one patient in the control group (1 of 30; 3.33%) discontinued follow-up. By the end of the follow-up in the tamsulosin group, six patients had reported nonserious adverse effects: four reported dizziness or nausea (4 of 30; 13.33%) and two male patients reported ejaculation disorders (2 of 30; 6.67%). The progress of patients through the different study phases is detailed in Figure 1.

The final analysis included 30 women and 27 men. The mean ± standard deviation for the age of all of the patients was 44.02 ± 12.79 years (range 20–76 years). Clinical characteristics of both groups are shown in Table 1. Based on the intergroup comparison, the ages of the groups and the proportion of patients by sex appeared to be similar (*p* = 0.080 and *p* = 0.230, respectively). There were no significant differences in renal stone laterality (*p* = 0.881), renal stone location (*p* = 0.836), or stone size (*p* = 0.591). Regarding the ESWL sessions, there was no evidence of differences among the groups in the duration of treatment, number of shocks, or total energy required (*p* = 0.941, *p* = 0.864, and *p* = 0.518, respectively). All of these results indicate that the groups were homogeneous with regard to clinical characteristics.

There were no complications during the ESWL sessions; nevertheless, complications and additional procedures during the follow-up by group are shown in Table 2. There were four events of symptomatic ureteral obstruction due to residual stones (4 of 57; 7.01%): two were in the control group (2 of 29; 6.90%) and two were in the tamsulosin group (2 of 28; 7.14%) (*p* = 0.971). Regarding the additional procedures used to resolve obstructive uropathy, there was no significant difference (*p* = 0.971). In the control group, one patient required a double-J stent and the other patient required a second ESWL session. In the tamsulosin group, both patients underwent laser endolithotripsy.

Overall, the mean ± standard deviation for stone size was 11.42 ± 4.52 mm (range 5–20 mm) in diameter. There was no significant difference in stone size between groups (*p* = 0.591). Regarding the stone-free rate, it was 50.88% (29 of 57) overall, 53.57% (15 of 28) for the tamsulosin group, and 48.27% (14 of 29) for the control group (*p* = 0.680); there was no significant difference in the stone-free rate between the groups. The estimated relative risk in favor of the tamsulosin group to achieve a stone-free status was 1.11 (95% CI 0.67–1.9). The estimated number needed to treat to achieve a single patient with renal stone-free status after eight weeks of ESWL adjuvant treatment with tamsulosin was 19.

Additionally, we identified 29 stones between 11 and 20 mm in diameter; in this case, the stone-free rate was 57.14% (8 of 14) for the tamsulosin group and 33.33% (5 of 15) for the control group (*p* = 0.198). The tamsulosin group had an apparent but not significantly higher stone-free rate among patients with renal stones of 11–20 mm in diameter.

## 5. Discussion

The adjuvant effectiveness of tamsulosin after ESWL therapy for single renal stone clearance is controversial.  Table 3 details the clinical features and results of diverse randomized studies included for review of this topic. As in our study, the studied dosage of 0.4 mg/day in the tamsulosin group was the common denominator for all of these studies, and the methodological differences were mainly identified in the study duration, stone location, stone size (mm) in diameter, and diverse methods used for residual fragment evaluation.

In the present randomized nonplacebo-controlled study, the results did not show a significant difference in the stone-free rate after a single ESWL session between the patients treated with tamsulosin and the patients in the control group (53.57% vs. 48.27%; *p* = 0.680) over eight weeks for patients with a single renal stone (located in the renal pelvis, upper calix, or middle calix) and with the stone size between 5 and 20 (mm) in diameter. We observed results similar to those described by De Nunzio et al. [17], who identified 58% stone-free rate in the tamsulosin group, 47% in the silodosin group, and 55% in the control group, with no statistically significant difference (*p* = 0.399) between patients treated over 21 days after a single ESWL session. In concordance, Ahmed et al. [18] also did not find a significant difference in the stone-free rate between patients in the tamsulosin group and the control group (78% vs. 69%, respectively, *p* = 0.108) in an up to 12-week study. In the same way as described in our study, they divided the patients into two categories (stone size ≤10 mm and stone size >10 mm), and they also found no differences in the stone-free rate by size, which matches our results.

We should note that the stone-free rates reported by Zaytoun et al. [19] were higher than those in our results. They described stone-free rates of 92% for the tamsulosin group, 90% for the doxazosin group, and 84% for the standard treatment group in a 12-week study. Similar to our study, these differences did not reach statistical significance; the higher values of free stone rates could be explained by the multiple ESWL sessions (average 2.07 sessions per patient). An additional placebo-controlled study conducted by Falahatkar et al. [20] reported stone-free rates of 71.4% and 60.5% among patients in the tamsulosin group and placebo group, respectively, in a 30-day study, a difference that was not statistically significant. They had higher stone-free values than those in our study. They only applied a single session of ESWL for the treatment of kidney stones, but they also included patients with ureteral stones.

In contrast with our findings, other authors have found a statistically significant improvement in the stone-free rate in patients treated with tamsulosin as an adjuvant to a single session of ESWL for the treatment of single kidney stones. The results of Gravina et al. [11] identified a significant difference in the success of the treatment between the tamsulosin group and the control group (78.5% vs. 60%; *p* = 0.037) in a study with a single session of ESWL and a treatment period of 12 weeks. When the authors stratified by stone size, a significant difference in the stone-free rate was observed only in those patients with stones greater than 10 mm in diameter (81% in the tamsulosin group vs. 55% in the control group, *p* = 0.009). Our study had lower stone-free rates than this study in both groups. An explanation for this might be that their study analyzed the presence of residual stones after ESWL by renal ultrasound, KUB plain X-ray, and/or excretory urography. This is in contrast with our study, wherein a CT scan was performed, a method that is considered more sensitive in the detection of small residual fragments. This may have been the cause of the lower stone-free rates described in our study.

Similar results were reported by Naja et al. [21], who described a statistically significant difference in favor of the tamsulosin group for the stone-free rate when they analyzed the results of the treatments three weeks after a single ESWL session (52.9% tamsulosin vs. 30.8% control, *p* = 0.016). However, when they evaluated the overall success at three months, there was no statistically significant difference (94.1% tamsulosin vs. 84.6% control, *p* = 0.14). During the three months of this study, multiple ESWL sessions were carried out (1 to 4 per patient). This study also evaluated the presence of residual stones with KUB plain X-ray and sometimes with ultrasound, and these facts could explain the high stone-free rate in both groups.

The study of Hussein et al. [13] reported a global stone-free rate of 73% evaluated by KUB plain X-ray and ultrasound at the end of four weeks in the tamsulosin group compared with 55% in the control group (*p* = 0.008), but the size of the renal stones was location limited (<25 mm for pelvic stones, <15 mm for upper calyceal and midcalyceal stones, and <10 mm for lower calyceal stones with favorable calyceal anatomy). These facts clearly influenced their favorable results. Similarly, the results of Bhagat et al. [15] showed a significant difference in the stone-free rate (96.6% tamsulosin vs. 79.3% control; *p* = 0.040); the difference remained significant for larger stones (11 to 24 mm) but not for small stones (6 to 10 mm), and the authors did not clearly specify the method used for evaluating the presence of residual fragments at the end of the study. In another placebo-controlled study, Vicentini et al. [12] found a significant difference in favor of treatment with tamsulosin after a single ESWL session in a 30-day study, but only for larger stones (10–20 mm), with a stone-free rate of 62% in the tamsulosin group vs. 26% in the placebo group (*p* = 0.024). Our results showed an apparent trend, although not statistically significant (*p* = 0.198), in favor of the tamsulosin group in the stone-free rate for larger stones (11–20 mm in diameter), and these results are consistent with the findings of the last two cited placebo-controlled studies. Our findings might have reached statistical significance if we had included more patients with larger stones.

In our study, tamsulosin treatment was generally well tolerated. Six patients (20%) reported nonserious adverse effects: four reported dizziness or nausea (13.33%) and two male patients reported ejaculation disorders (6.67%), but only for one patient (3.33%) was it necessary to suspend treatment due to symptomatic postural hypotension. These findings are consistent with those of other authors [11, 13, 15, 21] who described a similar low rate of adverse events. Their most frequently described adverse events were nausea, hypotension, ejaculation disorders, and diarrhea. In contrast, Zaytoun et al. [19] found a higher rate of adverse effects with tamsulosin (32%), mainly dizziness, nausea, headache, vomiting, and ejaculation disorders. These differences could be related to the longer treatment period (12 weeks) than that in our study (eight weeks).

Regarding complications, none of our patients experienced complications during the single ESWL procedure. However, during follow-up, four patients (7.01%) presented with acute urinary obstruction events due to residual stones that required auxiliary procedures for resolution of the obstruction. We did not find any differences regarding complications between the study groups (*p* = 0.971). These complication rates do not differ from those reported by other authors [18, 19]. However, other studies reported different complication-rate values. De Nunzio et al. [17] reported that 20% (12 of 60) of the patients had complications; 11 of them presented with acute pain requiring only analgesic treatment, and one patient underwent an additional procedure (ureteroscopy). The authors reported a statistically significant difference in the complication rate in favor of the tamsulosin group (*p* = 0.008). Additionally, Bhagat et al. [15] described the presence of residual stones in the ureter in 18 patients (31%), but only two patients from the placebo group (3.4%) required an intervention. Similarly, Vicentini et al. [12] reported complications in 29.9% of patients overall: 13.2% in the tamsulosin group, 11.4% in the nifedipine group, and 5.3% in the placebo group. However, only two patients (1.8%) required invasive procedures. It is important to highlight that all of the described complications during the follow-up appear to be independent of the use of tamsulosin, and this should be taken into account in the management of patients with single renal stones.

This study has certain limitations. First, the findings are based on a single-center study with a small number of patients (*n* = 60) with a single radiopaque kidney stone of 5–20 mm in diameter, and thus, interpretations should be made with caution. If there were more patients, tamsulosin could be observed to be significantly more effective. Second, some critics might suggest that the study lacks a placebo group. However, the identified homogeneity of the control group (standard treatment) and the tamsulosin group permitted valid intergroup comparisons. Last, confounding factors such as the stone size, location, and nutrition status (BMI) of patients could be controlled in a more comprehensive way. However, the strengths of the study include its prospective-controlled randomized design and the inclusion of a CT scan evaluation (at eight weeks after ESWL) made blindly by a trained urologist for a precise determination of the stone-free status in each patient.

## 6. Conclusions

Our findings suggest that tamsulosin as adjuvant treatment after a single ESWL session is well tolerated and safe, but it does not increase the stone-free rate in patients with a single radiopaque renal stone of 5–20 mm in diameter. Our results may support the use of tamsulosin with ESWL in the case of patients with a single radiopaque renal stone of 11–20 mm in diameter based on an apparent higher stone-free rate and a low rate of complications. Furthermore, multicenter, high-quality, randomized, and placebo-controlled trials evaluating the efficacy and safety of tamsulosin are necessary because their use remains controversial.

## Figures and Tables

**Table 1:** Clinical characteristics of the study population by group.

	Control group (*n* = 29)	Tamsulosin group (*n* = 28)	Intergroup comparison
Age (years)	47.14 (15.07)	40.79 (9.09)	*p* = 0.080^a^
Sex			*p* = 0.230^b^
Female, *n* (%)	13 (44.83)	17 (60.71)	
Male, *n* (%)	16 (55.17)	11 (39.29)	
Laterality			*p* = 0.881^b^
Right renal stone, *n* (%)	13 (44.83)	12 (42.86)	
Left renal stone, *n* (%)	16 (55.17)	16 (57.14)	
Location			*p* = 0.836^b^
Renal pelvis, *n* (%)	11 (37.93)	12 (42.86)	
Upper calix, *n* (%)	7 (24.14)	5 (17.86)	
Middle calix, *n* (%)	11 (37.93)	11 (39.29)	
Stone size (mm) in diameter	11.79 (4.87)	11.04 (4.18)	*p* = 0.591^a^
Duration of treatment (minutes)	54.28 (7.86)	54.54 (7.56)	*p* = 0.941^a^
Number of shocks (number)	3857.48 (389.72)	3863.36 (370.56)	*p* = 0.864^a^
Total energy (joules)	153.21 (29.98)	150.64 (32.74)	*p* = 0.518^a^

Unless otherwise indicated, values are given as mean (standard deviation). ^a^Mann–Whitney *U* test. ^b^Chi-square test.

**Table 2:** Complications and additional procedures during the follow-up by group.

	Control group (*n* = 29)	Tamsulosin group (*n* = 28)	Intergroup comparison
Obstructive uropathy, *n* (%)	2 (6.90)	2 (7.14)	*p* = 0.971^a^
Additional procedures			*p* = 0.971^a^
Laser endolithotripsy, *n* (%)	0 (0.0)	2 (7.14)	
Double-J catheter, *n* (%)	1 (3.45)	0 (0.0)	
Second ESWL, *n* (%)	1 (3.45)	0 (0.0)	

^a^Chi-square test.

**Table 3:** Clinical features and the results of the randomized studies included for review.

Source	Treatment arms, subjects	Dosage	Study duration	Single ESWL	Single stone (in diameter)	Unicenter study	Results (SFR)	Intergroup comparison	Residual fragment (in diameter) evaluation
De Nunzio et al. [17], 2016	Tamsulosin, *n* = 19	0.4 mg/day	21 d	Yes	Renal (5–20 mm)	Yes	58%	*p* = 0.399	(Not mentioned)
	Silodosin, *n* = 19	8 mg/day					47%		Ultrasound and
	Placebo, *n* = 22	--					55%		CT scan
Ahmed et al. [18], 2016	Tamsulosin, *n* = 123	0.4 mg/day	12 wk	Yes	Renal (≤20 mm)	No (four centers)	78%	*p* = 0.108	(<4 mm)
	Standard, *n* = 126	--					69%		KUB plain X-ray,
									ultrasound, and CT scan
Zaytoun et al. [19], 2012	Tamsulosin, *n* = 50	0.4 mg/day	12 wk	No (1 to 4)	Renal (≤20 mm)	Yes	92%	*p* > 0.050	(<3 mm)
	Doxazosin, *n* = 50	1–4 mg/day					90%		KUB plain X-ray and ultrasound
	Standard, *n* = 50	--					84%		
Falahatkar et al. [20], 2011	Tamsulosin, *n* = 70	0.4 mg/day	30 d	Yes	Renal or ureteral (4–20 mm)	Yes	71%	*p* > 0.050	(Not mentioned)
	Placebo, *n* = 71	Placebo					61%		KUB plain X-ray and ultrasound
Gravina et al. [11], 2005	Tamsulosin, *n* = 65	0.4 mg/day	12 wk	Yes	Renal (4–20 mm)	Yes	79%	*p* = 0.037	(≤3 mm)
	Standard, *n* = 65	--					60%		KUB plain X-ray, ultrasound, and urography
Naja et al. [21], 2008	Tamsulosin, *n* = 51	0.4 mg/day	3 mo	No	Renal (5–20 mm)	Yes	94%	*p* = 0.140	(≤3 mm)
	Standard, *n* = 65	--		(1 to 4)			85%		KUB plain X-ray and ultrasound
Hussein [13], 2010	Tamsulosin, *n* = 67	0.4 mg/day	4 wk	Yes	Renal (<25 mm) location limited	Yes	73%	*p* = 0.008	(<3 mm)
	Standard, *n* = 69	--					55%		KUB plain X-ray and ultrasound
Bhagat et al. [15], 2007	Tamsulosin, *n* = 29	0.4 mg/day	30 d	Yes	Renal (6–24 mm)	Yes	97%	*p* = 0.040	(<3 mm)
	Placebo, *n* = 29	--			Ureteral (6–15 mm)		79%		Not mentioned
Vicentini et al. [12], 2011	Tamsulosin, *n* = 38	0.4 mg/day	30 d	Yes	Renal	Yes	61%/62%	*p* = 0.118	(≤4 mm)
	Nifedipine, *n* = 35	20 mg/day			(5–20 mm)/		49%/60%	*p* = 0.024	KUB plain X-ray and ultrasound
	Placebo, *n* = 38	--			(10–20 mm)		37%/26%		
Maldonado-Valadez et al., 2021	Tamsulosin, *n* = 28	0.4 mg/day	8 wk	Yes	Renal	Yes	54%/57%	*p* = 0.680/	(≤4 mm)
	Standard, *n* = 29	--			(5–20 mm)/		48%/33%	*p* = 0.198	KUB plain X-ray and CT scan
					(11–20 mm)				

ESWL: extracorporeal shock wave lithotripsy; SFR: stone-free rate; KUB: kidneys, ureter, and bladder.

## Data Availability

All data underlying the findings are available on request to the corresponding author (edel.rodea@hraeb.gob.mx).
